# Characterisation of the correlation between standing lordosis and degenerative joint disease in the lower lumbar spine in women and men: a radiographic study

**DOI:** 10.1186/s12891-017-1696-9

**Published:** 2017-08-01

**Authors:** Kelvin J. Murray, Michael R. Le Grande, Arantxa Ortega de Mues, Michael F. Azari

**Affiliations:** 1 School of Health & Biomedical Sciences, RMIT University, PO Box 71, Bundoora, Melbourne, VIC 3083 Australia; 2 Australian Centre for Heart Health, Melbourne, Australia; 3 Faculty of Health, Deakin University, Melbourne, Australia; 4 Real Centro Universitario Escorial Maria Cristina, Madrid, Spain

**Keywords:** Lordosis, Lumbar region, Osteoarthritis

## Abstract

**Background:**

Degenerative joint disease (DJD) in the lumbar spine is a common condition that is associated with chronic low back pain. Excessive loading of lumbar joints is a risk factor for DJD. Changes in lumbar lordosis significantly redistribute the forces of weight-bearing on the facet joints and the intervertebral discs. However, the relationship between lumbar lordosis and DJD has not been characterized in men and women.

**Methods:**

We characterised the correlation between standing lumbar lordosis and DJD in standing radiographic images from 301 adult female and male chiropractic patients. DJD was rated using the Kellgren-Lawrence scale, and lordosis was measured using the Cobb angle. Linear and curvilinear correlations were investigated while controlling for age and sex.

**Results:**

We found a highly significant curvilinear correlation between lordosis and DJD of the lower lumbar spine in both sexes, but especially in women, irrespective of the effects of age. We found the effect size of lordosis on lower lumbar DJD to be between 17.4 and 18.1% in women and 12.9% in older men. In addition, lordosis of 65 (95% CI 55.3–77.7) and 68 (98% CI 58.7–73.3) degrees were associated with minimal DJD in the lower lumbar spine of women and men respectively, and were therefore considered ‘optimal’. This optimal lordotic angle was 73 (95% CI 58.8–87.2) degrees in older men.

**Conclusions:**

Both hypo- and hyper-lordosis correlate with DJD in the lumbar spine, particularly in women and in older men. These findings may well be of relevance to spinal pain management and spinal rehabilitation.

## Background

Degenerative joint disease (DJD) in the spine, also known as osteoarthritis (OA) affects approximately 80% of the population aged 40 and above [1]. It also has a complex association with chronic low back pain [2] and hence amounts to a significant health burden. Even though the etiology and pathogenesis of DJD is in need of further investigation, several risk factors for this condition have been identified. These factors include: abnormal or excessive joint loading [3, 4] for example as occurs in obesity [5] or excessive occupational standing or lifting; trauma; birth defects; and genetic predisposition [6]. Of these, excessive or abnormal joint loading is the most readily modifiable risk factor.

The primary postural curves of the spine (including the lumbar lordosis) provide optimal weight bearing by spinal joints [7, 8]. Changes in the magnitude of lumbar lordosis significantly change weight-bearing patterns in lumbar facet joints and intervertebral discs [9–13]. It is therefore plausible that significant changes from the ‘optimal’ degree of lumbar lordosis could overload spinal joints and influence development or progression of DJD. Should this be the case, a correlation would exist between increasing degrees of both hypo- and hyper-lordosis and DJD. The aim of this study was to investigate the presence and characteristics of such a correlation using standing radiographs in a large cohort of chiropractic patients. Moreover, we examined whether there were any sex differences in this putative correlation, as well as the magnitude of the optimal lordotic angle that corresponded with minimal DJD changes in the lower lumbar spine.

## Methods

### Radiographic images

Five hundred digital sets of standing A-P lumbo-pelvic and lateral lumbar radiographic images were randomly selected according to a computer-generated list and analyzed retrospectively from a pool of approximately 1300 radiographic series in the RMIT University Teaching Clinic archives. All radiographs were de-identified except for date of birth, gender and date of examination. One hundred ninety nine sets of images were excluded due to the following reasons: evidence of trauma; congenital developmental abnormalities such as transitional vertebrae; visible evidence of orthopedic surgery; leg length discrepancy; scoliosis; isthmic spondylolisthesis; fractures; tumors or any condition other than DJD; and poor image quality. The remaining 301 sets (156 women and 145 men) of radiographic images were then analyzed. The age range of subjects was 14 to 79 years. The vast majority of chiropractic patients in Australia present with spinal pain, particularly in the lower back [14]. In addition, the style of practice and case-mix of the RMIT University teaching clinic is representative of mainstream Australian chiropractic practice (unpublished data). However, clinical data were not sourced in order to avoid biasing the assessment of the radiographic images.

We ensured consistency of radiographic procedure and equipment by obtaining all the images from the one clinic. All standing A-P, and lateral radiographs were taken using standard positioning methods. The A-P lumbo-pelvic radiographs had been taken with the patient placed facing the X-ray source, with feet placed directly below the femur heads and lower limbs straight and without knee flexion. The lateral lumbar radiographs had been performed with the patient standing side on to the cassette, with arms crossed over the chest so that shoulder joints were flexed by approximately 30 degrees. This position has been shown to be suitable for measurements of lumbar lordosis [15].

### Radiographic measurements

Three hundred one digital sets of de-identified A-P lumbopelvic and lateral lumbar radiographs were used for this study. The images were opened with RadiAnt DICOM viewer software and enlarged to fit the screen of a 17-in. PC monitor. One investigator, an experienced clinician, scored all radiographic images for the presence and severity of DJD (KM) according to the Kellgren-Lawrence (K-L) criteria [16]. For ease of use, as has commonly been done in the literature [17], the K-L scores of 0 and 1 were deemed not to represent DJD and were given a score of 0, while the K-L scores of 2, 3 and 4 corresponded to mild (score of 1), moderate (score of 2) and severe (score of 3) DJD respectively in this study. Excellent intra-rater and inter-rater reliability has been reported by our group (Krippendorff’s alpha values above 0.91) for scoring DJD in the lumbar spine using this method [18]. Lumbar lordosis was measured using the commonly used ‘Gold standard’ which is the Cobb angle (from the superior endplate of L1 to the superior endplate of S1) [19]. Using RadiAnt DICOM viewer software, lines were drawn along the superior end plates of L1 and S1 and the angle between the two was measured, to derive the Cobb angle. All measurements were done by the same investigator to ensure consistency. In order to avoid bias, on each radiographic set DJD was evaluated first, before the Cobb angle was measured. A subset of 30 Cobb angle measurements were checked in a blinded fashion by a different investigator (MA) and were found to be extremely reliable (differences of up to 1 degree only). Pelvic incidence was not measured as many of the lateral images available did not provide a sufficient view of the hip joints. Sagittal inclination of the sacral base was measured but not presented in this manuscript as the reason for the lumbar lordosis observed was not the focus of this study.

### Statistical analysis

Descriptive statistics were used for age and lumbar lordosis (Cobb angle - CA) values. D’Agostino & Pearson Omnibus normality test was done using Graphpad Prism version 6.0 h, which revealed that CA and age values did not display normal distributions. Hence, CA values were raised to the power of two, and natural logs of age values were used. DJD scores for the entire lumbar spine (i.e. from L1/2 to L5/S1) were summed to generate a composite score for each individual that we termed Azari-Le Grande Degenerative Index (ALDI). Correlation between CA values and DJD scores for the lumbar spine (individual levels and ALDI scores) was investigated using both a linear and curvilinear (quadratic) model using SPSS version 22. We also combined the DJD scores of the lower 3 segments of the lumbar spine (i.e. L3/4, L4/5 and L5/S1) as well as the upper two segments (i.e. L1/2 and L2/3) for each individual (i.e. ALDI-Lower Lumbar and ALDI-Upper Lumbar values respectively) and investigated the same correlation using these composite scores. Care was taken to ensure that the curvilinear relationships were not in any way driven by outliers. Coefficient of determination (R-squared) was calculated to determine the proportion of variance in ALDI scores (for the entire lumbar spine) that could be explained by CA values. In addition, Standardized Beta-Coefficients were calculated for each group to more accurately determine the proportional effect of CAs on ALDI scores in different sexes and ages. Regression analyses were conducted for: age controlling for sex; sex controlling for age; and then within each age-sex subgroup controlling for age. The R-squared change values and F significance change values were then calculated. A significance level of *p* < 0.05 was used for type 1 errors.

## Results

**Fig. 1:**
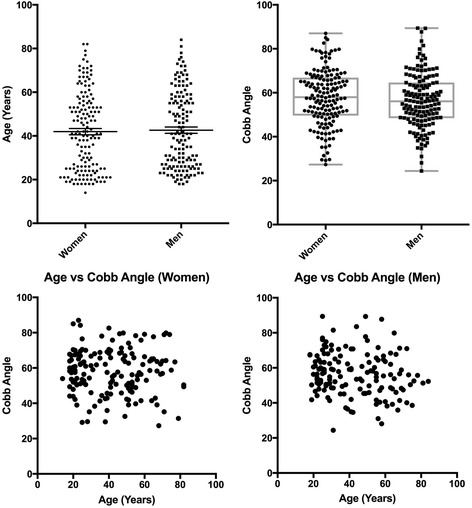
Distributions of age and Cobb angle (CA) values were comparable between men and women. With the exception of one case (a 14-year-old girl) all cases were adults who were primarily in their twenties or between 40 and 70 years of age. CA values represented near-normal distributions. Both age and CA values were comparable between the sexes

Before we interrogated the data, we examined the distributions of age and Cobb angle (CA) values in men and women in the cohort and found them to be similar (Fig. 1). Mean age values (± SD) for men and women were: 42.6 (± 17.4) and 41.9 (± 17.8) respectively. Similarly, mean lumbar lordosis [Cobb Angle] values (± SD) for men and women were: 56.53 (± 12.37) and 57.83 (± 12.72) respectively. Therefore, the cases could be directly compared across sex. We found no significant correlation between age and CA values in this study, since the spread of CA values as a function of age in both sexes was found to be random (Fig. 1).

We found significant quadratic (curvilinear) correlations between CA values and ALDI scores in all groups except for younger men where no correlation existed (Table 1). Correlations were stronger for women below the age of 40 (*p* = 0.042), and women aged 40 and over (*p* = 0.001) compared to corresponding groups of men (*p* > 0.05 and *p* = 0.036 respectively). We found that 19.7, 17.2 and 13% of cumulative DJD (ALDI) scores in younger women, older women, and older men respectively, could be explained by CA values (Table 1). The correlation between CA values and DJD scores was also interrogated at each individual level of the lumbar spine (Table 2). In women, at all five motion segments of the spine the correlation was significant with *p* values of 0.007 or lower. Correlations were either linear or quadratic in women and men at upper lumbar levels but quadratic at the lower three motion segments. Again, these correlations were far more significant in women. In fact, in men no correlation was found between CA values and DJD at the L5/S1 level. These findings indicated that abnormal (too little or too much) lordosis was a modest but significant driver of degeneration in the lumbar spine, and particularly in women.

**Table 1:**
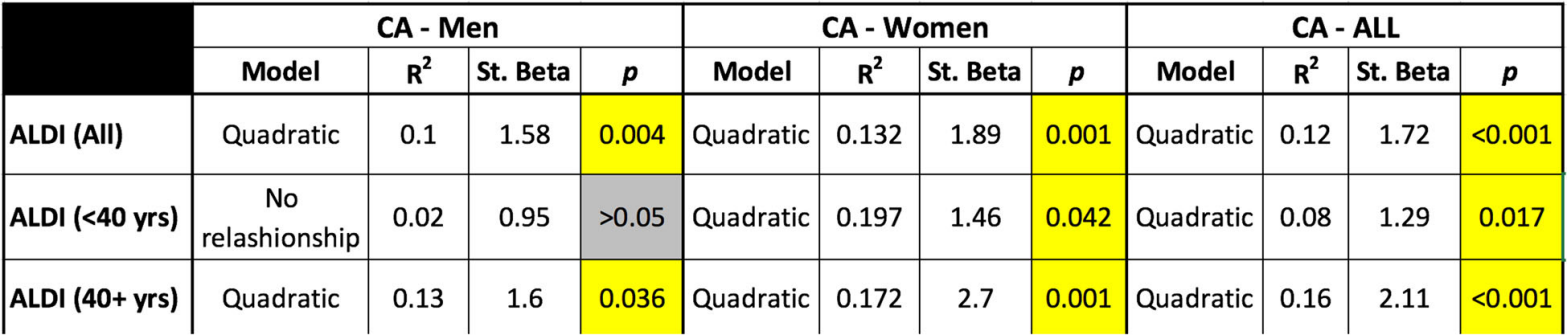
Significant quadratic correlations were found between CA and ALDI (Cumulative DJD) scores in all cases as well as 40 years plus cases. However, this correlation did not reach statistical significance in younger men

R-squared and Standardized Beta-Coefficient (St. Beta) values represent effect sizes and proportional effects of CAs on ALDI scores

**Table 2:**
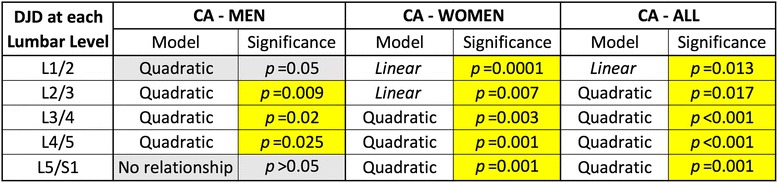
At upper lumbar levels (i.e. L1/2 and L2/3) the correlation showed either a quadratic (curvilinear) or a linear relationship

At lower levels however, the relationship was curvilinear, except at the L5/S1 level in men where no relationship was found. Again, correlations were stronger in women, in whom significantly lower *p* values were found at all lumbar levels

Since DJD is known to be much more common in the lower levels of the lumbar spine (i.e. L3/4, L4/5 and L5/S1 levels) the same correlation was investigated for ALDI scores that were calculated for these three motion segments together (ALDI-Lower Lumbar). This was also done for ALDI scores encompassing L1/2 and L2/3 segments (ALDI-Upper Lumbar) (Table 3). Again, significant quadratic correlations were found in all groups except in younger men. Highly significant correlations were found in women (both younger and older, and when controlled for age), and in those who were 40+. Effect sizes were calculated and expressed in percentage terms for the effect of CA on ALDI-Lower Lumbar values. These were: 12.6% in women; 7.2% in men; 15.4% in the older group; 3.1% in the younger group; 18.1% in older women; 17.4% in younger women; and 12.9% in older men. Together these data again suggested that the effect of lordotic angle on lower lumbar degeneration was highly significant while modest. This effect also increased with age and was much more pronounced in women.

**Table 3:**
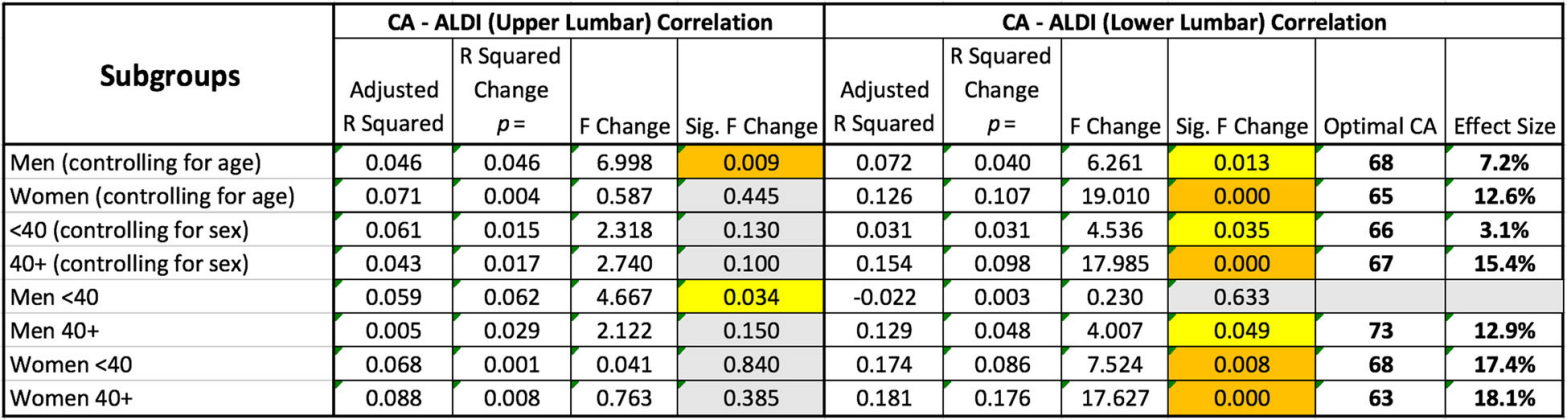
ALDI-Lower Lumbar scores correlated strongly with CA values except for younger men in which the correlation was found in the upper lumbar spine

Effect sizes (R-Squared values expressed as percentages) of CA values on ALDI scores ranged between 3.1% in younger people to 18.1% in older women. Significant correlations are shown in yellow, and highly significant correlations in orange. Non-significant associations are represented in grey

**Fig. 2:**
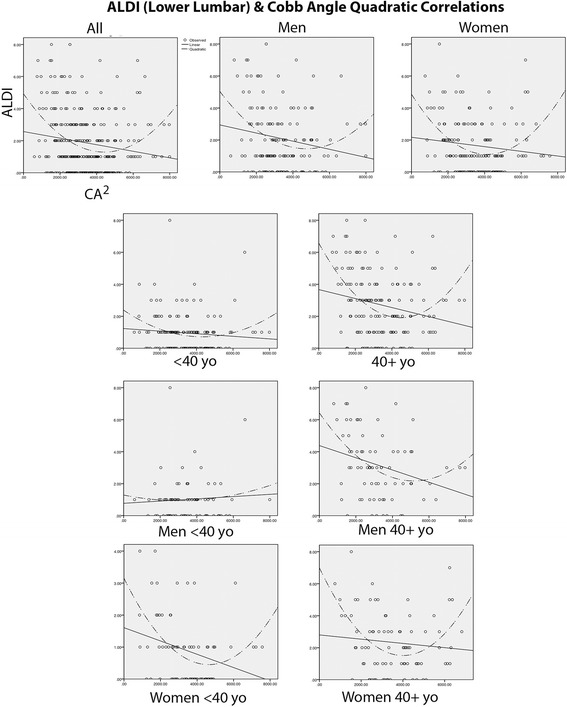
Curve plots of the correlations between CA values and ALDI-Lower Lumbar scores revealed the CA values that corresponded to minimal DJD in this region of the spine. They also demonstrated that ALDI scores increased equally in relation to hypo- and hyper-lordosis in women (whether young or old), but in men they increased much more sharply in relation to hypo-lordosis and only in the older age group

We then plotted the ALDI-Lower Lumbar correlation results to determine ‘optimal’ lordotic angles that were associated with minimal DJD in the lower lumbar spine (Table 3). Quadratic correlations meant that increasing deviation in either direction (hypo-lordosis or hyper-lordosis) of these optimal values was associated with increased incidence or severity of DJD in the lower lumbar spine. The optimal CA values were found to be: 65 (95% CI 55.3–77.7) for women; 68 (95% CI 58.7–73.3) for men; 67 (95% CI 56.1–77.9) in the older group; 66 in the younger group; 63 (95% CI 51.3–74.7) in older women; 68 (95% CI 52.6–83.4) in younger women; and 73 (95% CI 58.8–87.2) degrees in older men. These graphs also allowed visual inspection of the shape and steepness of the curve for each of these correlations (Fig. 2). All groups (except younger men) showed steeper rises in ALDI-Lower Lumbar scores in relation to decreasing CA values (hypolordosis). In all the groups studied (except in young men), hypolordosis showed a slightly stronger correlation with lower lumbar DJD. It should also be noted that in older men this correlation was much stronger for hypo-lordosis than for hyper-lordosis. While these differences were of note, it was clear that lordotic abnormalities in both directions significantly correlated with lower lumbar degeneration.

## Discussion

It is important to determine the ‘ideal’ or ‘optimal’ sagittal alignment of the lumbar spine in order to prevent or manage overloading of intervertebral discs and facet joints that may contribute to DJD. It has long been known that decreased lordosis increases the load on the intervertebral discs and increased lordosis increases the load on the facet joints [9, 10]. More importantly, it is known that increased pelvic incidence (PI), which increases lumbar lordosis, correlates with DJD of the facet joints [20–22], which in turn is associated with low back pain [23]. Increased PI is also associated with wedging of intervertebral discs, spondylolysis and spondylolisthesis [24]. In addition, it has been reported that disc herniation in older patients occurs at higher and lower levels of the lumbar spine with decreased and increased lordosis respectively [25].

The most reliable ‘normal range’ for lumbar lordosis, using postural radiographs, has been reported as 48–78 degrees [21, 22, 26]. The heterogeneity of methods used to measure lumbar lordosis in the literature has resulted in various ranges of proposed ‘normal’ values [21, 22]. This wide range of ‘normal’ values is also partly due to the fact that lumbar lordosis is driven primarily by the variation in the position-independent sagittal alignment of the pelvis (PI) [27, 28] which varies widely between individuals. Nevertheless, postural changes that alter the lumbar lordosis within this ‘normal’ range are likely to significantly change the load distribution of compressive forces over the spinal joints and lead to DJD. Lumbar lordosis can also be modulated by exercise [29] and postural habits [30]. This makes lumbar lordosis a potential therapeutic target for exercise rehabilitation and ergonomic intervention. Nevertheless, to date, the correlation between lordosis and DJD of the lower lumbar spine has not been characterized in detail. Unlike most studies on the subject that have relied on CT-scan measurements of lordosis in the supine position that do not represent lordosis under weight-bearing conditions, we used standing postural (weight-bearing) radiographic images for this purpose. Even more importantly, to our knowledge, this is the first study using standing lumbar images, to define the optimal lordotic angle corresponding to minimal degenerative change in the lumbar spine of both women and men in a primary care setting. It is significant to note that while we found mean lordotic values in our sample to be 56.53 and 57.83 in men and women respectively, the corresponding optimal values were 68 and 65 degrees. This may well indicate that the magnitude of lumbar lordosis is not optimal in most people.

Consistent with previous reports, we found age not to correlate with lumbar lordosis [31]. While genetics play a significant role in the development of spinal DJD [6, 32], so do postural and environmental factors. For instance, the female lumbar spine is morphologically suited to increased lordosis [33], and in young women decreased lumbar lordosis correlates with disc degeneration [34]. This has also been recently shown in men [20]. Similarly, Increased or prolonged loading of the intervertebral disc is thought to contribute to its degeneration through compromising its nutrient supply [35–37]. Consistent with this, is evidence that hypolordosis following spinal fusion surgery is associated with subsequent DJD of the adjacent segment [38].

We found the effect size of lordosis on lower lumbar DJD (ALDI-Lower Lumbar) to be between 17.4 and 18.1% in women and 12.9% in older men. These effect sizes while relatively modest are highly significant, particularly that lordosis is modifiable, unlike other DJD risk factors such as genetics. We identified for the first time that 65 and 68 degrees of lumbar lordosis, in women and men respectively, were associated with minimal DJD in the lower lumbar spine. In older men, this optimal value was 73 degrees. Deviation from these optimal values in the form of either hypo-lordosis or hyper-lordosis correlated with increased incidence and/or severity of DJD in this region of the spine that is commonly affected by DJD. Our findings are consistent with the notion that the lumbar spine is under optimal weight-bearing conditions at these defined lordotic angles. They may also advocate for rehabilitation strategies that attempt to restore or maintain these lordotic angles in order to prevent or retard the progression of DJD.

There is a report of the absence of a relationship between lordosis and DJD in the lumbar spine [39]. However, this was a small study of women only in a tertiary care setting and hence may not be representative of the general population. Our results together with the balance of the available literature, support the notion that lumbar hypolordosis is associated with spinal DJD. Additionally, given sexual dimorphism in the anatomy of the lumbar vertebrae and intervertebral discs [40], the sex differences identified in our study are not surprising. Even age-related changes in trabecular patterns in lumbar vertebrae show sexual dimorphism [41]. Therefore, it is important for future studies on this subject to also interrogate sex differences.

There are several limitations to this study. Whilst weight-bearing spinal radiographs allow accurate measurement of lumbar lordosis, CT and MRI scans allow superior evaluation of degenerative changes in facet joints and intervertebral discs respectively. The second limitation is these changes were not correlated with clinical information about low back pain, occupation, or body-mass-index. However, this was intentional so that the clinical information would not bias the assessment of the radiographic findings. We found a robust correlation in this study between lumbar lordosis and DJD. However, these results need to be replicated by others in different populations and settings before generalizable statements can be made about this association. In addition, ideally, to investigate cause-effect relationships between lumbar lordosis and lumbar DJD, as well as low back pain, a large longitudinal cohort study needs to be conducted.

Nevertheless, lumbar lordosis is modifiable using pelvic tilting exercises [42]. There is also recent RCT evidence that lumbar lordosis rehabilitation can reduce chronic low back pain [43]. Given our findings and these reports, we propose that targeted rehabilitation exercises that attempt to maintain or restore the ‘optimal lordotic angle’ may have clinical utility in perhaps delaying the progression of DJD in the lumbar spine. This approach would clearly need to be individualized as the extent to which lumbar lordosis is amenable to change is dependent on the individual patient’s pelvic incidence as well as the severity of existing DJD.

## Conclusions

We report here that DJD in the lower lumber spine is closely correlated with deviations of lumbar lordosis from approximately 65 degrees in both women and men from a large sample of adult chiropractic patients. A cause-effect relationship remains to be demonstrated. However, based on a developing body of evidence, our results and the understanding of clinical biomechanical principals, it can reasonably be hypothesized that deviations from an optimal lordotic angle in the lumber spine would compromise optimal weight bearing conditions on spinal joints and lead to the development of DJD. This notion is also supported by the lack of a correlation between age, and hyper- or hypo- lordosis, discounting degenerative age-related changes as a confounding variable. While these results need to be replicated in other adult populations, they may have important implications in prevention and treatment of lumbar spinal DJD. In addition, the case-mix of the patients presenting to this teaching clinic is similar to that of mainstream chiropractic practices in Australia, in that the vast majority of patients present with low back pain and neck pain. Hence, these results may well have implications for prevention, treatment and rehabilitation of low back pain.

## Abbreviations

ALDI: Azari-Le Grande degenerative index
CA: Cobb angle
DJD: Degenerative joint disease
K-L: Kellgren and Lawrence
OA: Osteoarthritis
